# Bisphenol A exposure triggers endoplasmic reticulum stress pathway leading to ocular axial elongation in mice

**DOI:** 10.3389/fmed.2023.1255121

**Published:** 2023-09-07

**Authors:** Junhan Chen, Shin-ichi Ikeda, Longdan Kang, Kazuno Negishi, Kazuo Tsubota, Toshihide Kurihara

**Affiliations:** ^1^Laboratory of Photobiology, Keio University School of Medicine, Tokyo, Japan; ^2^Department of Ophthalmology, Keio University School of Medicine, Tokyo, Japan; ^3^Department of Ophthalmology, The First Hospital of China Medical University, Shenyang, China; ^4^Tsubota Laboratory, Inc., Tokyo, Japan

**Keywords:** bisphenol A, endoplasmic reticulum stress, ATF6, PERK, sclera, myopia

## Abstract

**Background:**

Ocular axial elongation is one of the features of myopia progression. Endoplasmic reticulum (ER) stress-associated scleral remodeling plays an important role in ocular axial elongation. Bisphenol A (BPA) is one of the most common environmental pollutants and is known to affect various human organs through ER stress. However, whether BPA exerts an effect on scleral remodeling remains unknown. The purpose of this study was to determine the effect of BPA on the development of myopia and scleral ER stress.

**Methods:**

BPA was administered by intraperitoneal injection. 4-PBA was administered as an endoplasmic reticulum stress inhibitor by eye drops. Refraction and axial length were measured by refractometer and SD-OCT system. Western blot was performed to detect the expression level of ER stress-related proteins.

**Results:**

BPA-administered mice exhibit axial elongation and myopic refractive shift with endoplasmic reticulum stress in the sclera. BPA administration activated scleral PERK and ATF6 pathways, and 4-PBA eye drops attenuated ER stress response and suppressed myopia progression.

**Conclusion:**

BPA controlled axial elongation during myopia development in a mouse model by inducing scleral ER stress and activation of the PERK/ATF6 pathway. 4-PBA eye drops as ER stress inhibitor suppressed BPA-induced myopia development.

## 1. Introduction

The incidence of myopia is rapidly increasing worldwide, particularly in East Asia (1). The refractive status is determined by the balance of the refractive power of the cornea and lens as well as the axial length of the eye, which is the result of an uncoordinated contribution of ocular components to the overall ocular structure (2). Among these factors, axial length (AL), one of the main determinants of myopia, has received extensive attention in related studies (3).

The sclera plays an important role in controlling the shape and size of the eyeball (4). Previous studies have shown that scleral remodeling is regulated by multiple factors such as genetics and the environment (4). The matrix remodeling that occurs in the sclera during myopia development results in changes in biomechanical properties, which are critical for the increase in axial length that promotes myopia (5). However, the mechanism underlying scleral remodeling during myopia remains to be further elucidated. Recent studies have demonstrated that endoplasmic reticulum (ER) stress plays an important role in scleral remodeling in a form-deprivation myopia model in guinea pigs and a lens-induced myopia model in mice (6, 7). Activating transcription factor 6 (ATF6) is an ER transmembrane transcription factor with a mechanism for sensing ER stress and responding via translocation to the Golgi apparatus (8). Protein kinase RNA (PKR)-like ER kinase (PERK), mediated by phosphorylation of eukaryotic translation initiation factor 2 (eIF2α), is the major protein responsible for attenuated mRNA translation under ER stress (9). The canonical ER stress sensor proteins ATF6 and PERK are activated under ER stress to regulate the sclera and axial length (6). Moreover, 4-phenylbutyric acid (4-PBA), classified as a chemical chaperone, has been recognized as an inhibitor of ER stress (10). Numerous studies have substantiated the effectiveness of 4-PBA in mitigating ER stress across diverse cellular contexts, resulting in enhanced cell viability and functionality (11, 12). Notably, 4-PBA has been validated as an inhibitor capable of impeding myopia progression through its ability to diminish ER stress within the scleral tissue (6).

Bisphenol A (4,4-isopropylidenediphenol, BPA) is a common organic compound widely used in the production of various plastics and resins. Because of its wide range of uses, BPA has become one of the most widely produced industrial compounds worldwide (13). BPA has been detected as a potential health risk factor in a range of aquatic systems, wildlife, and humans as a potential health risk factor (14). In the ocular environment, BPA exposure can exacerbate hypertensive oculopathy in rat models (15). In addition, BPA-induced ER stress is associated with various pathological processes in the liver, nervous, and reproductive system (16–20). At the same time, the relationship between environmental pollution and myopia is receiving attention (21). However, substantive research on whether BPA affects ER stress in the ocular tissues, especially the sclera, is still lacking.

In this study, we explored the effects of BPA on the development of eye axial length in mice and investigated the mechanism of BPA exposure on the sclera from the perspective of ER stress.

## 2. Materials and methods

### 2.1. Materials

BPA was purchased from FUJIFILM Wako Pure Chemical Corporation (Osaka, Japan). Oil of corn (23–0320-5) purchased from Sigma was used as a vehicle. 4-Phenylbutyric acid (4-PBA) was purchased from Cayman Chemical (MI, USA; Catalog #11323). Relevant antibody sources and dilution multiple are listed in Table 1.

**Table 1:** The list of antibodies for western blot.

Name	Dilution ratio	Company	Catalog
ATF6	1:1000	Bio Academina	73–505
p-IRE1α	1:1000	GeneTex	GTX132808
IRE1α	1:1000	Cell Signaling Technologies	#3294
p-eIF2α	1:1000	Cell Signaling Technologies	#3398
eIF2α	1:1000	Cell Signaling Technologies	#5324
β-actin	1:5000	Cell Signaling Technologies	#3700

### 2.2. Animal administration

Male C57BL6J mice were housed in standard transparent cages in a temperature (24 ± 2°C) and humidity (40–60%) controlled clean room under a 12-h light–dark cycle. Animals had free access to a standard rodent diet and water throughout the experimental period.

All animal experiments in this study were approved by the Animal Experimental Committee of Keio University and adhered to the Institutional Guidelines on Animal Experimentation at Keio University, ARVO Statement for the Use of Animals in Ophthalmic and Vision Research, and Animal Research: Reporting of *In Vivo* Experiments (ARRIVE) guidelines for the use of animals in research.

The mice (3 weeks old) were randomly divided into two groups to detect whether BPA could induce ER stress in the sclera: the BPA and control groups. In the BPA group, 100 mg/kg BPA was administered daily for 14 days by intraperitoneal (IP) injection, and the mode of administration and dose were determined based on previous studies (22, 23). Mice in the control group were injected with the same volume of vehicle.

In the 4-PBA inhibition of endoplasmic reticulum stress experiments, since data from previous study showed that 4-PBA eye drops alone did not significantly affect axial length and refraction (6), mice were randomly divided into three groups: control, BPA, and BPA + 4-PBA. Mice in the BPA group received 100 mg/kg BPA and PBS eye drops once daily for 14 days. BPA + 4-PBA group was injected 100 mg/kg BPA and 4-PBA (2% solution) eye drops, once daily for 14 days. The control group received the same volume of vehicle and PBS eye drops.

### 2.3. Ocular biometric measurements

Refractions were obtained using a refractometer (Steinberis Transfer Center, Tübingen, Germany) subsequent to the induction of general anesthesia in mice through intraperitoneal injection of midazolam (40 μg/100 μL; Sandoz, Tokyo, Japan), medetomidine (7.5 μg/100 μL; Orion, Espoo, Finland), and butorphanol tartrate (50 μg/100 μL; Meiji Seika Pharma, Tokyo, Japan). The measurement of axial length (AL) was conducted utilizing a spectral domain-optical coherence tomography (SD-OCT) system (Envisu R4310, Leica), specifically designed for mice, in accordance with established methodologies outlined in prior study (24).

### 2.4. Western blot

After anesthesia as described above, mice were euthanized by cervical dislocation followed by enucleation of eyes for further tissue isolation. Sclera samples were homogenized in RIPA buffer (50 mM HEPES (pH 7.5), 150 mM NaCl, 1% NP-40, 50 mM NaF, 10 mM β-glycerophosphate, 5 mM benzamidine, 0.1% sodium deoxycholate 1 mM EDTA, 1 mM Na3VO4, and 1 mM PMSF) containing Halt protease inhibitor cocktail (ThermoFisher Scientific, USA). The protein concentration was measured using a bicinchoninic acid (BCA) protein assay and adjusted with Laemmli sample buffer (Nacalai Tesque). Extracted protein samples were resolved by SDS-PAGE, then transferred to PVDF membranes (Merck Millipore, MA, USA), blocked with Blocking One (Nacalai Tesque, Tokyo, Japan). After that, the membrane was incubated overnight at 4°C with IRE1 alpha, IRE1, phosphor-eIF2α, eIF2α, ATF6 and β-actin antibodies at 4°C. The corresponding secondary antibody (1:10000) was incubated with the membrane at room temperature for 1 h. The SuperSignal West Femto Maximum Substrate (Thermo Fisher Scientific) was used for visualization. SDS-PAGE was performed on 10% acrylamide gels using protein size markers (MagicMark XP Western Protein Standard, Thermo Fisher Scientific).

### 2.5. Statistical analysis

Independent sample Student’s two-tailed *t*-test and analysis of variance (ANOVA) with Fisher’s least significant difference (LSD) *post hoc* test were performed using GraphPad Prism 9 to determine the statistical significance of the comparisons. Image J (version 1.52v; NIH) was used for histogram analysis of the western blots. *p* < 0.05 was considered statistically significant.

## 3. Results

### 3.1. BPA induce ocular axial elongation

**Figure 1:**
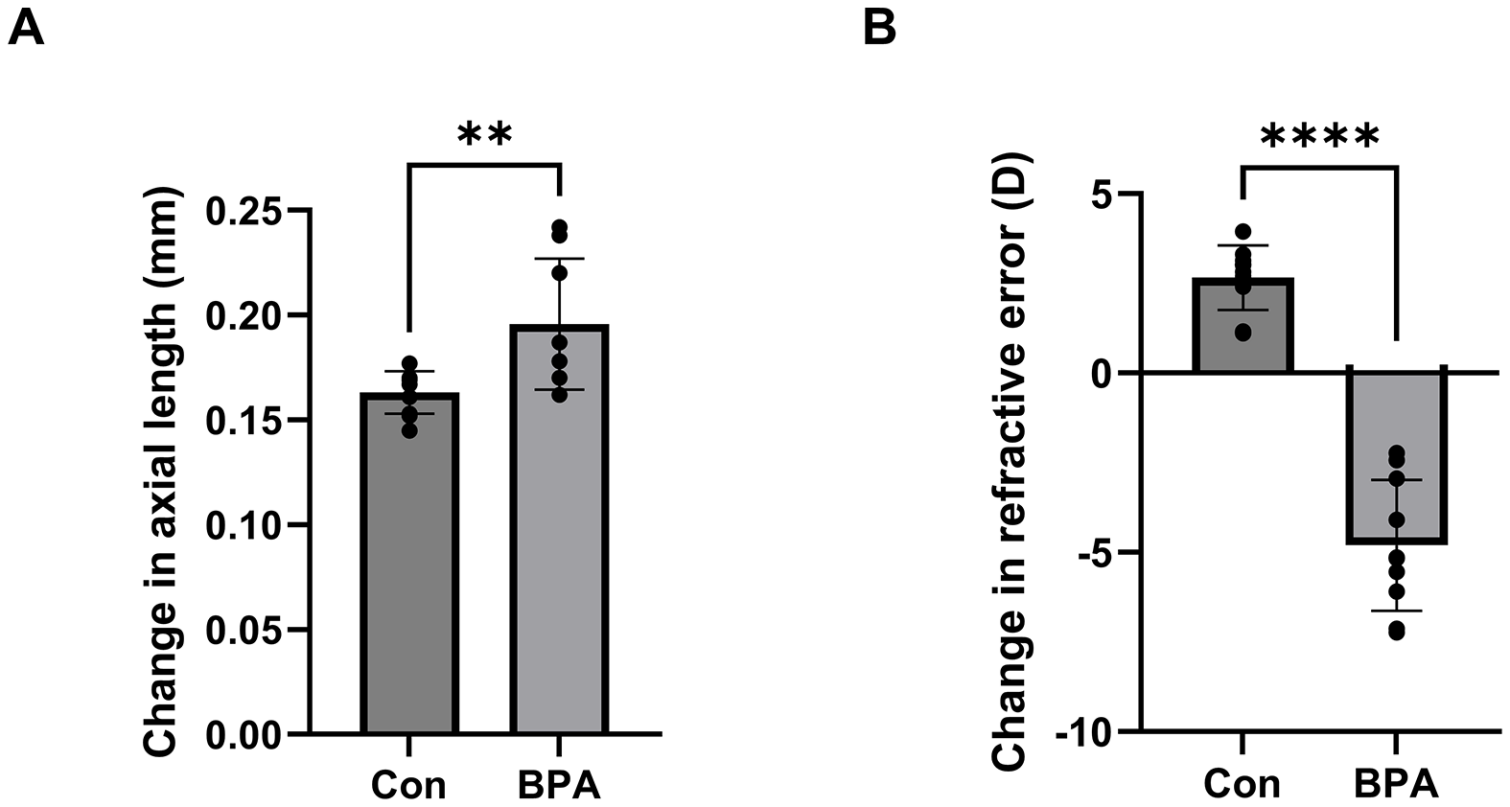
BPA induced axial elongation and myopic refraction shift. **(A)** Change in axial length during 2-week BPA administration in C57BL6J mice (*n* = 10). Con: control group with corn oil administration; BPA: BPA group, BPA was administered 100 mg/kg BPA daily. Student’s two-tailed *t*-test, **p < 0.01. The values are presented as mean ± SD. **(B)** Change in refractive error during 2-week BPA administration in C57BL6J mice (*n* = 10). Con: control group with corn oil administration; BPA: BPA group, BPA was administered 100 mg/kg BPA daily. Student’s two-tailed *t*-test, ****p < 0.0001. The values are presented as mean ± SD.

To determine whether BPA could induce ocular axial elongation and myopia development, BPA was administered for 2 weeks. Changes in axial length and refraction were detected using SD-OCT and refractometer systems between the oil- and BPA- administration groups. Mice of BPA administration group showed axial elongation (*p* < 0.05) compared with oil administration group (ΔAL means ± SD Control:0.163 ± 0.010, BPA:0.198 ± 0.028 mm) (Figure 1A). At the same time, a significant myopic shift (*p* < 0.0001) occurred compared to control eyes (ΔRF means ± SD Control:2.66 ± 0.86, BPA: −4.80 ± 1.73 D), which were typical features of myopia development (Figure 1B). In addition, we also analyzed the changes in corneal thickness and retinal thickness by OCT between the oil- and BPA- administration groups but failed to find significant differences (Supplementary Figures S1A,B).

### 3.2. BPA induce ER stress in sclera

The sclera is a key tissue that controls the axial length of the eye (4). Previous studies by our group demonstrated that ER stress occurs in the sclera and leads to matrix remodeling and axial elongation in myopia (6). To explore the effect of BPA on scleral ER stress, we measured the changes in the expression levels of scleral endoplasmic reticulum stress-related proteins in mice exposed to BPA (100 mg/kg/d, 14 days).

**Figure 2:**
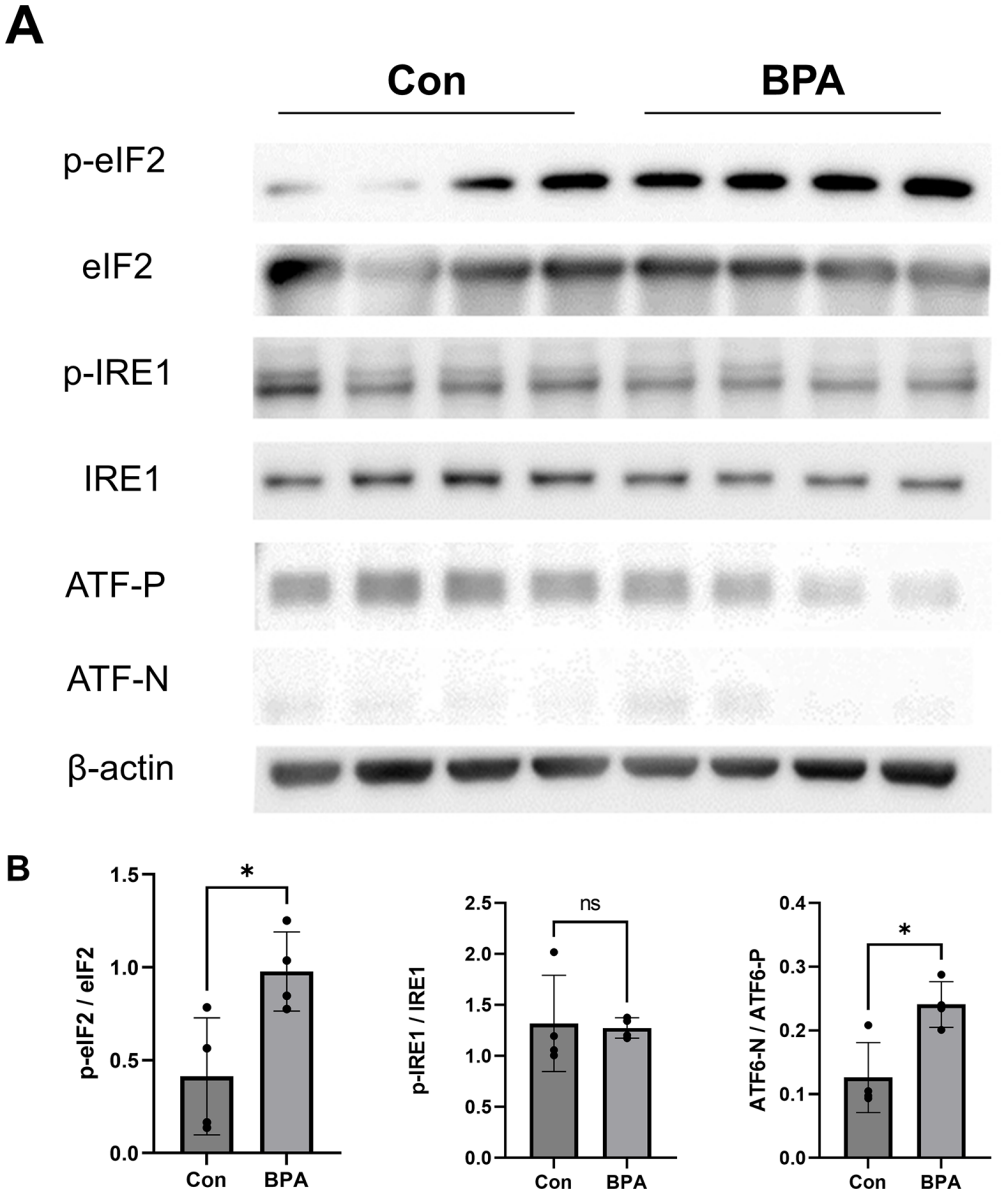
ER sensor protein activation by BPA administration. **(A)** Western blot results showed ER sensor protein activation (phosphorylation levels of IRE1, PERK, eIF2α, and the ATF6 precursor and cleaved form of ATF6). Con: control group with corn oil administration; BPA: BPA group, BPA was administered 100 mg/kg BPA daily. **(B)** Densitometric quantification of the blot in **(A)** using ImageJ. Con: control group with corn oil administration (Blue); BPA: BPA group, BPA was administered 100 mg/kg BPA daily (Red). Student’s two-tailed *t*-test, *p < 0.05, NS, Not Significant. The values are presented as mean ± SD.

Scleral samples were collected to assess the expression of the ER stress-related proteins IRE1, eIF2, and ATF6 (Figure 2A). BPA administration group showed higher phosphorylation levels of eIF2, which is a downstream factor of the PERK pathway in the sclera (Figure 2B). Simultaneously, the ratio of cleaved activated ATF6 (ATF6-N) to full-length ATF6 (ATF6-P) was higher in the BPA group (Figure 2B). This is consistent with the conclusions of our previous study that the PERK and ATF6 pathways are involved in scleral remodeling and ocular axial elongation (6). However, there was no difference in the p-IRE1/IRE1 ratio between the control and BPA-treated groups (Figure 2B). This result is similar to previous reports in some organs, where BPA activated the PERK and ATF6 pathways but failed to affect the IRE1 pathway (25).

### 3.3. Attenuation of scleral ER stress to suppress BPA induced myopia

**Figure 3:**
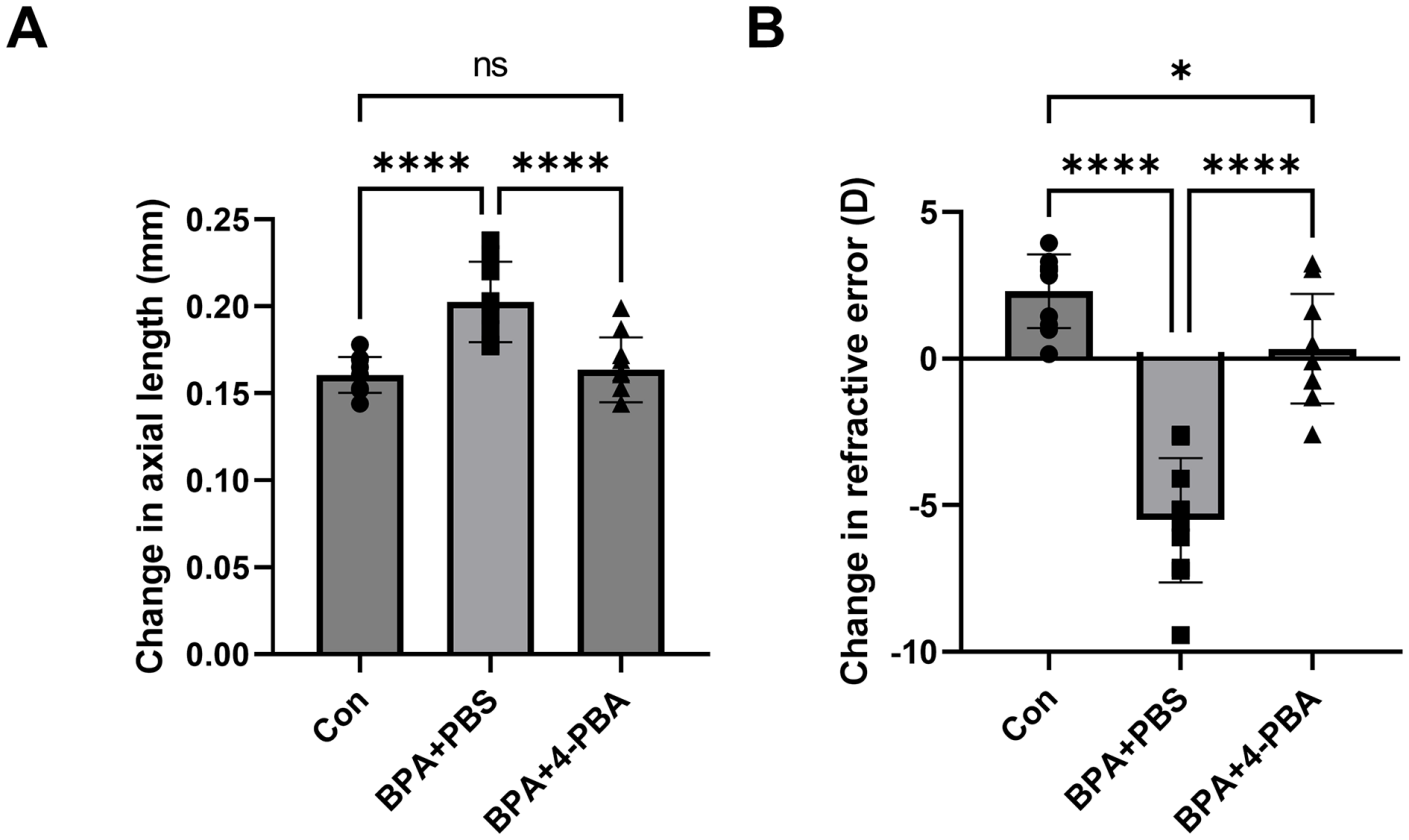
Effect of ER stress inhibitors on BPA-induced myopia development. **(A)** Change in axial length during 2-week BPA administration with 4-PBA eye drops in C57BL6J mice (*n* = 10). Con: control group with corn oil administration, PBS eye drops; BPA + PBS: group with BPA administration, PBS eye drops. BPA + 4-PBA: group with BPA administration, 4-PBA eye drops (2% solution in PBS). One-way ANOVA with Fisher’s LSD *post hoc* test, ****p < 0.0001, NS: Not Significant. The values are presented as mean *±* SD. **(B)** Change in refractive error during 2-week BPA administration with 4-PBA eye drops in C57BL6J mice (*n* = 10). Con: control group with corn oil administration, PBS eye drops; BPA + PBS: group with BPA administration, PBS eye drops. BPA + 4-PBA: group with BPA administration, 4-PBA eye drops (2% solution in PBS). One-way ANOVA with Fisher’s LSD *post hoc* test, *p < 0.05, ****p < 0.0001. The values are presented as mean ± SD.

To determine whether scleral ER stress is one of the main factors in BPA-induced axial elongation and myopia development, 4-phenylbutyric acid (4-PBA) was used to attenuate scleral ER stress. During BPA administration, 4-PBA (2% solution in PBS) was administered as eye drops, and changes in axial length and refraction error were compared between the PBS eye drop administration group and the oil control group. Compared with the BPA + PBS group, the BPA + 4-PBA group showed a shorter change in the axial length of the eye (ΔAL means ± SE Control:0.161 ± 0.004, BPA + PBS:0.203 ± 0.009, BPA + 4-PBA:0.164 ± 0.006 mm) (Figure 3A). Similarly, 4-PBA-administered mice reduced the myopic shift in refraction (ΔRF means ± SE Control:2.30 ± 1.19, BPA + PBS: −5.51 ± 2.00, BPA + 4-PBA:0.34 ± 1.77 D) by 2 weeks (Figure 3B).

**Figure 4:**
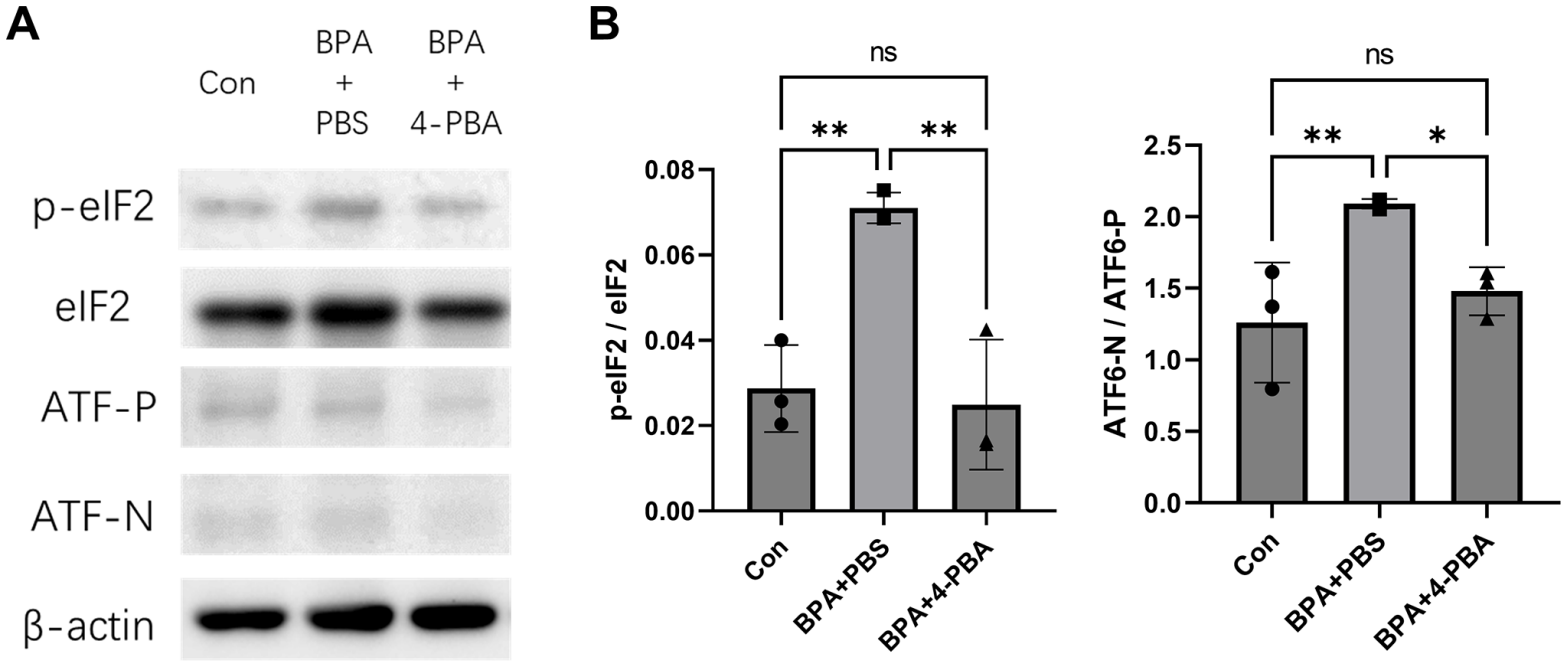
Effect of ER stress inhibitors on BPA-induced scleral ER stress. **(A)** Western blot showed effect of 4-PBA on BPA-induced scleral ER stress (phosphorylation levels of IRE1, PERK, eIF2α, and the ATF6 precursor and cleaved form of ATF6). Con: control group with corn oil administration, PBS eye drops; BPA + PBS: group with BPA administration, PBS eye drops. BPA + 4-PBA: group with BPA administration, 4-PBA eye drops (2% solution in PBS). **(B)** Densitometric quantification of the blot in **(A)** using ImageJ. Con: control group with corn oil administration, PBS eye drops; BPA + PBS: group with BPA administration, PBS eye drops. BPA + 4-PBA: group with BPA administration, 4-PBA eye drops (2% solution in PBS). One-way ANOVA with Fisher’s LSD *post hoc* test, *p < 0.05, **p < 0.01, NS, Not Significant. The values are presented as mean ± SD.

To further verify the effect of 4-PBA on scleral ER stress, the expression levels of PERK (assessed by eIF2) and ATF6 pathway-related ER stress markers were detected using western blotting (Figures 4A,B). BPA administration activated mouse scleral eIF2 phosphorylation and the ATF6 pathways in the BPA + PBS group. In contrast, the 4-PBA eye drop-treated group showed a lower ER stress response in the sclera. The western blotting results corresponded to the refraction and axial elongation results. BPA administration activated the PERK and ATF6 pathways, whereas 4-PBA eye drops attenuated ER stress and reduced myopic shifts in refractive and axial elongation.

## 4. Discussion

Substantial evidence suggests that environmental BPA may adversely affect human health (26–28). A recent study pointed out that BPA administration can affect the eye, but extensive research in this area is lacking (15). At the same time, East Asia, a major producer of BPA (29), is facing a myopia epidemic (30), which means that it will be interesting to further explore the effects of BPA on the eye. In the current study, we observed that BPA administration group showed axial elongation and a myopic refraction shift. Simultaneously, BPA induced ER stress in the sclera, especially via the PERK and ATF6 pathways.

The sclera plays an important role in controlling the size of the eyeball (31). Axial elongation, accompanied by scleral matrix remodeling, is a hallmark of myopia progression (32). It was recently reported that hypoxia, biomechanical stress, and ER stress may be related to scleral remodeling, indicating that there may be multiple factors involved in the regulation of the sclera during the process of myopia development (33, 34). Furthermore, our previous research illustrated that endoplasmic reticulum stress within the sclera could wield a substantial influence over the expression of ECM proteins. This eventuality transpires through the activation of both the PERK and ATF6 pathways, culminating in the subsequent restructuring of scleral collagen (6). BPA affects extracellular matrix remodeling in various organs; however, its effect on the sclera has not been reported (35–37). Our results confirm that BPA may affect scleral remodeling. Similarly, BPA exposure reduced the repair function of myofibroblasts and their ability to successfully remodel after myocardial infarction (35). Together, these findings highlight the importance of scleral remodeling as a potential research target in myopia.

Accumulation of unfolded proteins in the ER activates ER stress sensor proteins such as PERK, ATF6, and IRE1 (38). ER stress is associated with various physiological and pathological conditions, including matrix remodeling and fibrosis (39–42). ER stress promotes nuclear pulposus cell apoptosis and disc degeneration by affecting extracellular matrix homeostasis (39). During pulmonary fibrosis, ER stress can affect profibrotic effector pathways, including apoptosis, differentiation, and inflammatory signaling (40). In Schmid metaphyseal chondrodysplasia, ER stress occurs in chondrocytes and activates the PERK, ATF6, and IRE1 pathways, whereas IRE1 is not involved in the short-bone-length phenotype (42). Simultaneously, scleral matrix remodeling is recognized as an important factor in myopia development (4, 31). Given that BPA can induce ER stress in multiple organs (16, 18, 19, 43), it would be valuable to investigate the association between BPA, scleral ER stress, and myopia.

**Figure 5:**
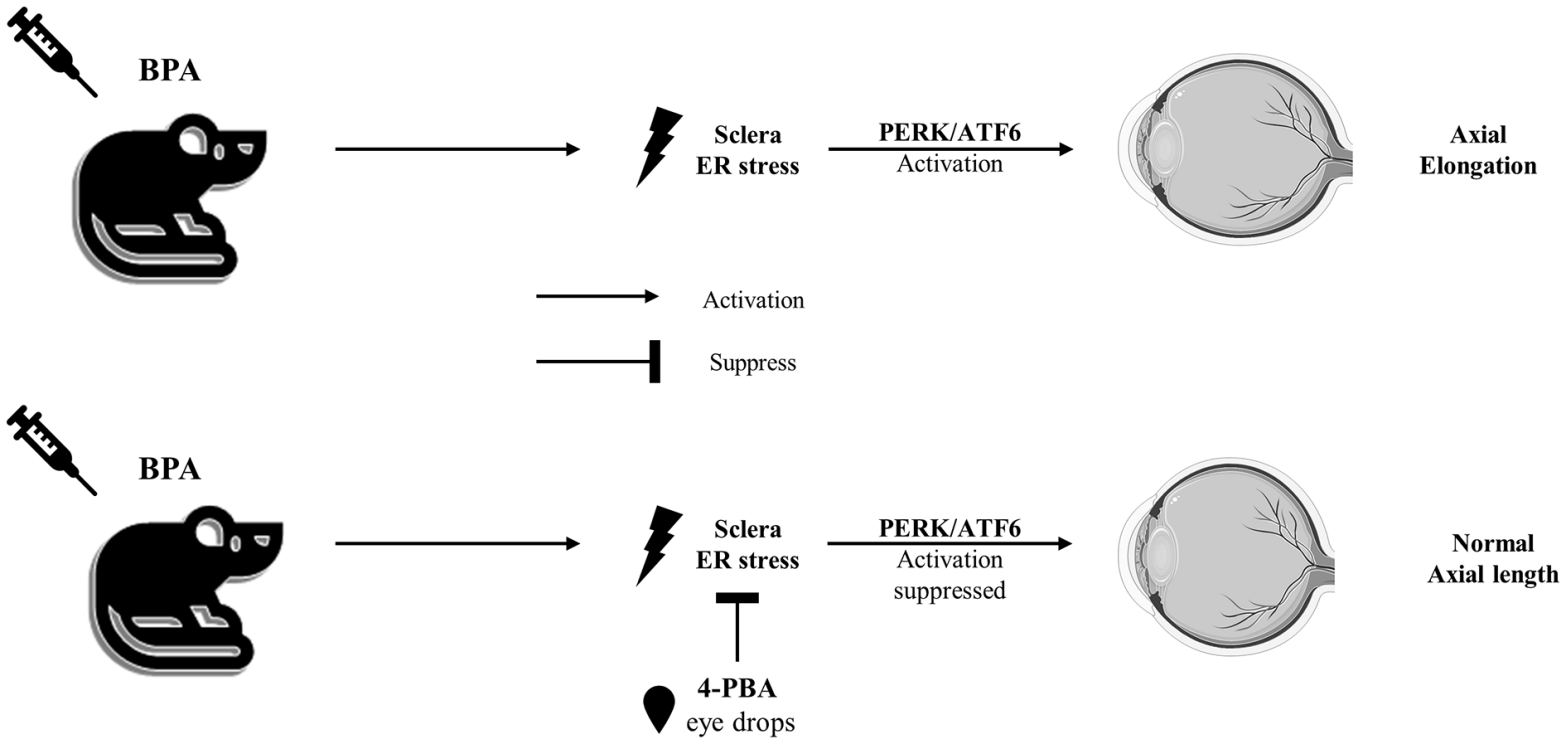
The sketch map of the pathway mediated by BPA to induce axial elongation and suppressed by 4-PBA.

In our experimental mouse model, BPA administration upregulated the expression of ER stress-related proteins in the sclera, particularly in the PERK and ATF6 pathways (Figure 5). According to previous report, lens-induced myopia caused ER stress in the sclera rather than the retina in mouse models, and the induction of scleral ER stress was sufficient to induce changes in eye axial length (6). Consistent with our previous report (6), myopia development was associated with scleral ER stress, as myopia development was not induced in the control group (administered corn oil), whereas BPA induced the upregulation of ER stress-associated proteins, axial elongation, and myopic refractive change. These results suggested a cause-and-effect relationship between ER stress and BPA-induced myopia in mice. Furthermore, BPA-induced progression of myopia was attenuated by the ER stress inhibitor 4-PBA. This inhibitory effect was demonstrated by changes in the expression of ER stress-related proteins, reduction in axial growth, and refractive changes in mice. However, it is worth noting that there are differences in the patterns of human exposure to BPA and in animal models. The United States Environmental Protection Agency (EPA) has established a reference dose (RfD) of 0.05 mg/kg body weight (BW)/day for BPA in humans, which is derived from adverse effects observed in rats exposed to 1,000 mg/kg BPA (44). Future additional experiments would be advantageous in order to investigate the potential long-term impacts of BPA on the sclera.

Although existing results point to ER stress as the main pathway for BPA-induced myopia, it must be pointed out that there may be other pathways that play a role in this process. Several research studies have indicated a connection between the adverse effects caused by BPA and the dysregulation of autophagy (45). In the process of BPA’s effect on myocardial remodeling, BPA-induced inflammation is considered a potential factor and MMP-2 is considered a response molecule to inflammation (35). In myopia research, it has also been suggested that inflammation may play a role in sclera remodeling (46). However, another study demonstrated that although the upregulation of scleral MMP-2 induces a myopic refractive shift, no meaningful change in ocular axial length is observed (47). In addition, some studies have indicated that BPA directly regulate the expression of MMP-9 to mediate extracellular matrix remodeling (48). The role of MMP-9 in the myopic sclera requires further study. On the other hand, BPA has been shown to have estrogen-like effects (49). Although the expression of estrogen receptor alpha protein was not detected in human male and female sclera samples in previous reports (50), it would be interesting to compare the effects of BPA on the eyes of mice of different sexes in the future.

Moreover, a noteworthy aspect to consider pertains to the exposure pathway of BPA to the human body, particularly the ocular tissue. It is imperative to underscore that while our study used intraperitoneal administration for experimental precision, the actual exposure of ocular tissues to BPA predominantly occurs from contact lenses and containers of eye drops (51, 52). In forthcoming research endeavors, the adoption of low-dose topical administration via eye drops would be instrumental in more faithfully emulating the genuine routes of BPA exposure encountered in real-life context.

In conclusion, our study demonstrated that BPA administration induces scleral endoplasmic reticulum stress and results in axial elongation of the mouse eye. 4-PBA inhibited the progression of BPA-induced myopia. Furthermore, the PERK and ATF6 axes were the main pathways activated during BPA-induced ER stress.

## Data availability statement

The raw data supporting the conclusions of this article will be made available by the authors, without undue reservation.

## Ethics statement

The animal study was approved by the Animal Experimental Committee of Keio University. The study was conducted in accordance with the local legislation and institutional requirements.

## Author contributions

JC: Data curation, Formal analysis, Investigation, Writing – original draft. S-iI: Data curation, Investigation, Methodology, Project administration, Writing – original draft. LK: Data curation, Methodology, Writing – review & editing. KN: Supervision, Writing – review & editing. KT: Funding acquisition, Supervision, Writing – review & editing. TK: Conceptualization, Funding acquisition, Investigation, Project administration, Supervision, Writing – review & editing.

## Funding

This work is supported by Support for Pioneering Research Initiated by the Next Generation (SPRING) by Japan Science and Technology Agency (JST) to JC and Grants-in-Aid for Scientific Research (KAKENHI) from the Ministry of Education, Culture, Sports, Science and Technology to, S-iI (20K09834), and TK (21H03096). This work is also supported by the Grant for Myopia Research from Tsubota Laboratory, Inc. This work was also supported by Japan Agency for Medical Research and Development (AMED) under Grant Number JP22 gm1510007 to TK.

## Conflict of interest

KT reports his position as CEO of Tsubota Laboratory, Inc., Tokyo, Japan, a company producing myopia-related devices.

The remaining authors declare that the research was conducted in the absence of any commercial or financial relationships that could be construed as a potential conflict of interest.

## Publisher’s note

All claims expressed in this article are solely those of the authors and do not necessarily represent those of their affiliated organizations, or those of the publisher, the editors and the reviewers. Any product that may be evaluated in this article, or claim that may be made by its manufacturer, is not guaranteed or endorsed by the publisher.
