# Supplementary material for: Bisphenol A exposure triggers endoplasmic reticulum stress pathway leading to ocular axial elongation in mice
Source: Front Med (Lausanne). 2023 Sep 7;10:1255121. doi: 10.3389/fmed.2023.1255121 (PMC10517050; doi:10.3389/fmed.2023.1255121)
Supplement: Supplementary file 1 [file Data_Sheet_1.docx]

Supplementary Material

## Supplementary Figures


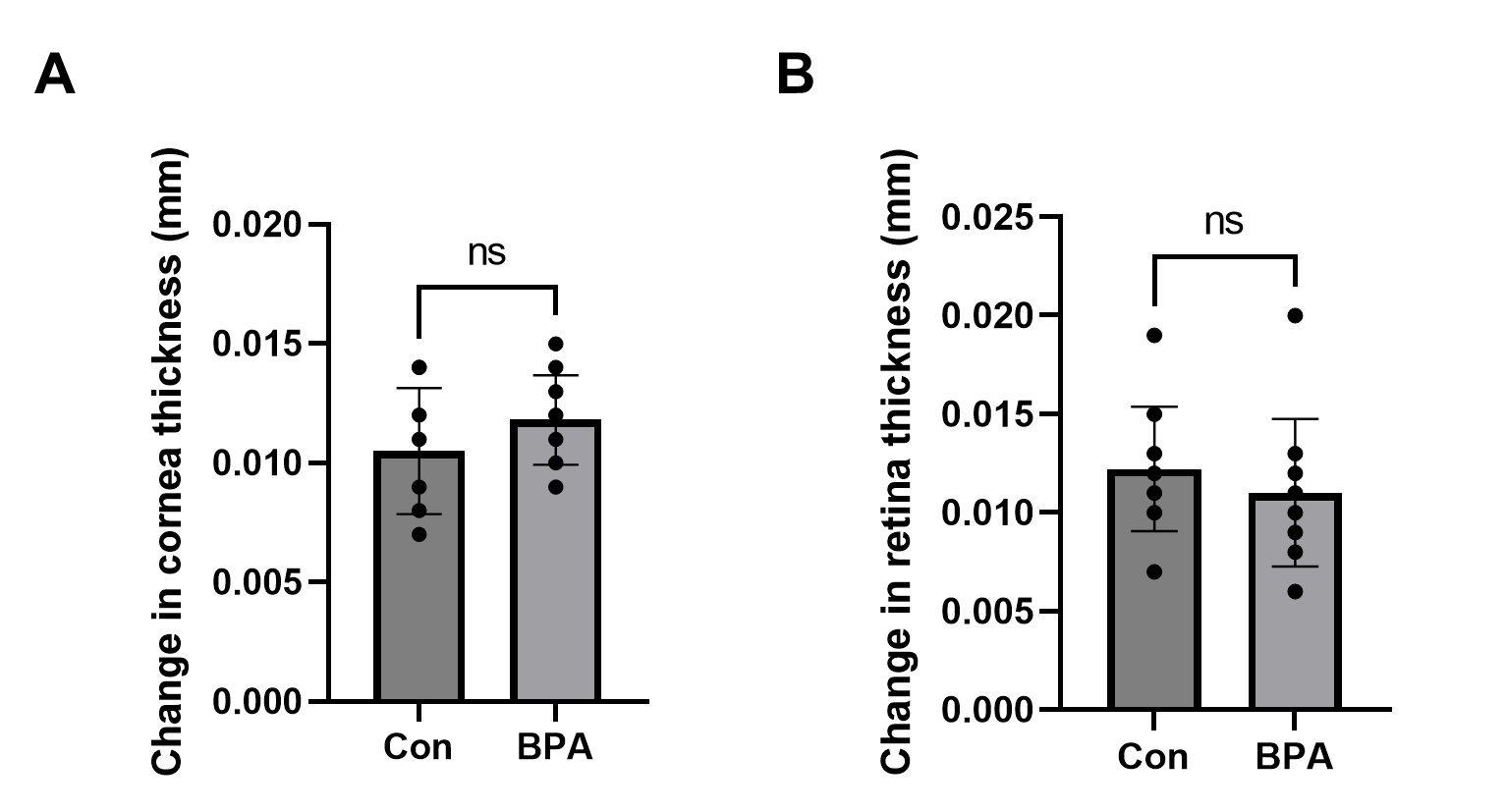


**Supplementary Figure 1.** (A) Change in cornea thickness during 2-week BPA administration in C57BL6J mice (n=10). Con: control group with corn oil administration; BPA: BPA group, BPA was administered 100 mg/kg BPA daily. Student’s two-tailed t-test, NS: Not Significant. The values are presented as mean ± SD. (B) Change in refractive error during 2-week BPA administration in C57BL6J mice (n=10). Con: control group with corn oil administration; BPA: BPA group, BPA was administered 100 mg/kg BPA daily. Student’s two-tailed t-test, NS: Not Significant. The values are presented as mean ± SD.
